# The role of vitamin D on rotator cuff tear with osteoporosis

**DOI:** 10.3389/fendo.2022.1017835

**Published:** 2022-11-18

**Authors:** Gejun Liu, Wenjun Li, Liyun Zhang, Chao Zhou, Ruijun Cong

**Affiliations:** ^1^ Department of Orthopedics, The Shanghai Tenth People's Hospital of Tongji University, Shanghai, China; ^2^ Department of Medical Iconography, Shanghai Changzheng Hospital, Naval Medical University, Shanghai, China; ^3^ Department of Orthopaedics, Yinshanhu Hospital of Wuzhong District, Suzhou, China

**Keywords:** rotator cuff, osteoporosis, nutrient levels, LASSO, biomarkers

## Abstract

**Backgrounds:**

Osteoporosis (OP) is an important risk factor for rotator cuff tears (RCTs). However, the relationship and mechanism between rotator cuff injury and osteoporosis are unclear. Therefore, to investigate association between rotator cuff injury and osteoporosis, and find clinical characteristics, bone mineral density, bone metabolism markers, and nutrient levels in rotator cuff injury patients with or without osteoporosis.

**Methods:**

One hundred and four cases of patients (RCTs, n=32; RCTs-OP, n=72) who underwent rotator cuff injury and need arthroscopic rotator cuff repair between June 2021 and February 2022, along with the diagnosis of osteoporosis were identified from the dual-energy X-ray bone density screening(DXA). The outcome measure includes clinical characteristics, bone mineral density, bone metabolism markers, vitamins, and amino acids. Multivariable logistic regression analysis was applied to build a predicting model incorporating the feature selected in the least absolute shrinkage and selection operator regression model. Discrimination, calibration, and clinical usefulness of the predicting model were assessed using the C-index, calibration plot, and decision curve analysis. Internal validation was assessed using bootstrapping validation.

**Results:**

OP with RCTs has a lower level of in 25-vitD, osteocalcin (OCN), serum Ca2+, ornithine, diaminocaproic_acid but the high level of Vitamin_B12, PTH, Vitamin_D3,γ_aminobutyric_acid, Vitamin_C and Vitamin_E than RCTs patients without OP. Predictors contained in the prediction nomogram included lumber T score, femur T score, Niacin_B3, and vitamin D, reflecting the combined effect of vitamins on RCTs-related OP progression. The model has good discriminative ability with a C-index of 0.938(95% CI:-1.83-1.39) and good scaling ability. The high C-index value of 0.95 is still achievable with range validation. Analysis of decision curves showed that non-adherence is clinically useful when intervention decisions are at the 14% probability limit of non-adherence.

**Conclusion:**

This study supports the hypothesis that lumber T score, femur T score, Niacin_B3, and Vitamin D are valuable prognostic biomarkers on RCTs related OP progression.

**What is known about the subject:**

It is found that vitamin D are valuable prognostic biomarkers, reflecting the combined effect of vitamins on RCTs related OP progression.

**What this study adds to existing knowledge:**

These findings also highlight that nutrients condition such as vitamins and amino acids of patients provide a new understanding of the development of RCTs.

## Introduction

Rotator cuff tears (RCTs) is one of the diseases that severely affect the movement of the shoulder joint in patients, with an incidence of about 15-20% in patients aged 60 years, about 26-30% in patients aged 70 years, and 36-50% in patients aged 80 years (1). With the accumulation of factors such as increasing exercise patterns or aging, the incidence of RCTs is increasing significantly, which bringing serious pain and loss of function (2, 3). RCTs also carry a huge financial burden, bringing about $3-5 million a year in the United States (4). The probability of re-tearing after a rotator cuff tear is about 20-40%, which also suggests that there is a risk of poor healing of tendons embedded in the bone (5–8). However, there are still significant deficiencies in poor rotator cuff repair capabilities and current limitations of surgery and injection therapy (9). Therefore, it is of great significance for further study of the mechanism of rotator cuff injury.

Vitamins and amino acids have been reported to improve tendon and muscle mass by inhibiting collagen metabolism and reducing tendon flexibility (10–12). In a mouse model, vitamin D (VitD) prevented local decrease in bone mineral density (increased roughness of the proximal humerus) and improved rotator cuff tendon healing (12). Low serum vitamin B12 levels are associated with degenerative rotator cuff tear (13). In addition, leucine ameliorated tenotomy-induced muscle atrophy by inhibiting autophagy and possibly activating the PI3K/AKT/mTOR pathway in rotator cuff injury (14). However, there are no established biologic factors of vitamin and amino acids to improve tendon to bone healing. Therefore, it is important to find specific treatment strategies or drugs that can improve patient outcomes in large-scale randomized controlled trials.

Osteoporosis is a serious health problem that seriously affected the health of the elderly population, affecting 200 million people worldwide (15–17). In joint-related diseases, osteoporosis was a dependent risk of in patients who required total knee repalcement (18). The diagnosis of osteoporosis is mainly based on dual-energy X-ray absorptiometry (DXA) analysis, and a T value of bone density less than -2.5 is diagnosed as osteoporosis (19, 20). Differences in recovery rates have led many studies to investigate factors that may contribute to structural failure with rotator cuff, such as age, smoking, tear size, fatty infiltration, and metabolic syndrome (21, 22). In addition, osteoporosis is a other risk factor in the prognosis of rotator cuff injury and is thought to significantly increase the risk of re-tearing after rotator cuff repair (10, 23–25). However, there is a lack of in-depth research on the close relationship between osteoporosis and rotator cuff injury. For example, whether the bone quality will affect the loosening of the anchor or cause the anchor to fall out at the beginning of fixation, which will lead to the failure of rotator cuff repair surgery (26, 27).

Considering that the relationship between full-thickness tears of the rotator cuff and osteoporosis also increases significantly with age, this further indicates that osteoporosis has obvious significance for rotator cuff injuries (28). In this paper, the central thesis is 1)to analyze whether there is a correlation between bone mineral density (BMD) in the lumbar spine, humerus, and femoral head of patients with rotator cuff injury and its relationship with clinical features. In addition, 2) we also developed an efficient and simple predictive tool to assess the risk factors of vitamins, amino acid levels, and markers of bone metabolism in patients with rotator cuff injuries combined with osteoporosis.

## Materials and methods

This study is a single-center, prospective cross-sectional study. This study was approved and approved by the ethics committee and the institutional review board (IRB) of our Hospital. All patients were informed and signed an informed consent form. According to the CONSORT guidelines, these studies also comply with the Declaration of Helsinki.

### Patients

The study recruited 104 patients from our hospital who required rotator cuff surgery at the author’s institution in June 2021 and February 2022. We included patients with full-thickness rotator cuff tears treated only arthroscopically and confirmed by arthroscopy, and patients with postoperative rotator cuff integrity confirmed by magnetic resonance imaging (MRI).

The study included patients who were at least 50 years old and who passed one of the above RCT tests. Exclusion criteria were: (1) previous shoulder surgery; (2) previous known shoulder lesions; (3) wheelchair or bedridden persons; (4) active tumor pathology, neurological syndrome, peripheral neuropathy, Obesity, moderate to severe cognitive impairment, and other chronic diseases; history of fragility hip fracture. In addition, rotator cuff injuries due to trauma or sudden strenuous exercise are excluded. Patient demographics and other characteristics were recorded, including age, gender, location, time of surgery, smoking status, hypertension, and other cardiac conditions (Representative Rotator Cuff Injury Video 1-3).

### Study outcomes

Osteoporosis is diagnosed primarily based on total hip or hip BMD T-score ≤-2.5 and 1 or more moderate or severe vertebral fractures or 2 or more mild vertebral fractures; o Total femoral neck or femur BMD T-score of -2.0 or lower, and 2 or more moderate-to-severe vertebral or proximal femur fractures (29). The lumbar spine and proximal femur were then assessed for BMD by dual-energy X-ray absorptiometry (lunar or holographic).

Magnetic resonance imaging (MRI) assessed tendon integrity in both groups. Coronary, sagittal and axial t2wi echo resonances were performed in all patients. Complete cuff rupture was defined as a defect in the extension of the supraspinatus tendon from the joint capsule to the articular surface. Teardrop shapes are classified according to DeOrio and Cofield (30): Tears are divided into three groups according to the length of their largest diameter: small (0-10 mm), medium (10-30 mm) or large (30-50 mm). According to Goutallieri et al., there is fatty infiltration in the muscles that rotate the cuff. Registration Classification: Class 0, no fatty infiltration; Class 1, oily streaks; Class 2, more muscle than fat; Class 3, muscle and fat; Class 4, more muscle than fat, less fat (31).

Serum concentrations of the bone-turnover markers β-isomer of C-terminal telopeptide of type I collagen (CTX; LabCorp), 1,25-dihydroxyvitamin D(3) (1,25-vitD, Covance), Alkaline phosphatase (ALP, Covance), Calcitonin, Osteocalcin (OCN, Covance) were measured in a subgroup of 104 patients. Serum amino acid levels and vitamin levels are also measured. Identification of amino acids was performed by comparing retention times with those from a standard calibration mixture. The concentrations were determined by the conventional calculation of areas under the peaks. Quality control of each batch of amino acid analyses was affected by the inclusion of an aliquot of deproteinate obtained from a pooled plasma. Serum vitamins were detected by an HPLC device equipped with an electrochemical detector (32).

### Statistical analysis

All data including the demographic and the metrics are expressed using mean± standard deviation and count(%). Statistical analysis is carried out using R software (Version 3.1.1; https://www.R-project.org ) and SAS version 9.3 (SAS 9.3, SAS Institute, Cary, NC).

The LASSO (Least Absolute Shrinkage and Selection Operator) method was used to reduce multivariate data and select risk factors for osteoporosis at rotator cuff rupture. The training set uses non-zero LASSO regression coefficients. Multiple logistic regression analysis was then performed on selected features in the LASSO regression model to create a predictive model. The characteristics odds ratio (OR) with 95% confidence interval (CI) and p-value were taken into account. The statistical significance levels are two-tailed. Sociodemographic variables were included in the model with a p-value less than 0.05, while disease- and treatment-related variables were included. A calibration curve was drawn to evaluate the calibration of the prediction nomogram, and the Harrell c index was measured to quantify the discriminative strength of the prediction nomogram.Whether to perform decision curve analysis to identify clinically useful prediction nomograms to quantify net benefit. The diagnostic efficacy of the clinical factor model was assessed on the basis of the appropriate ROC curve (AUC) of the training and validation toolkit (33–37).

## Results

### Patients’ characteristic

**Figure 1:**
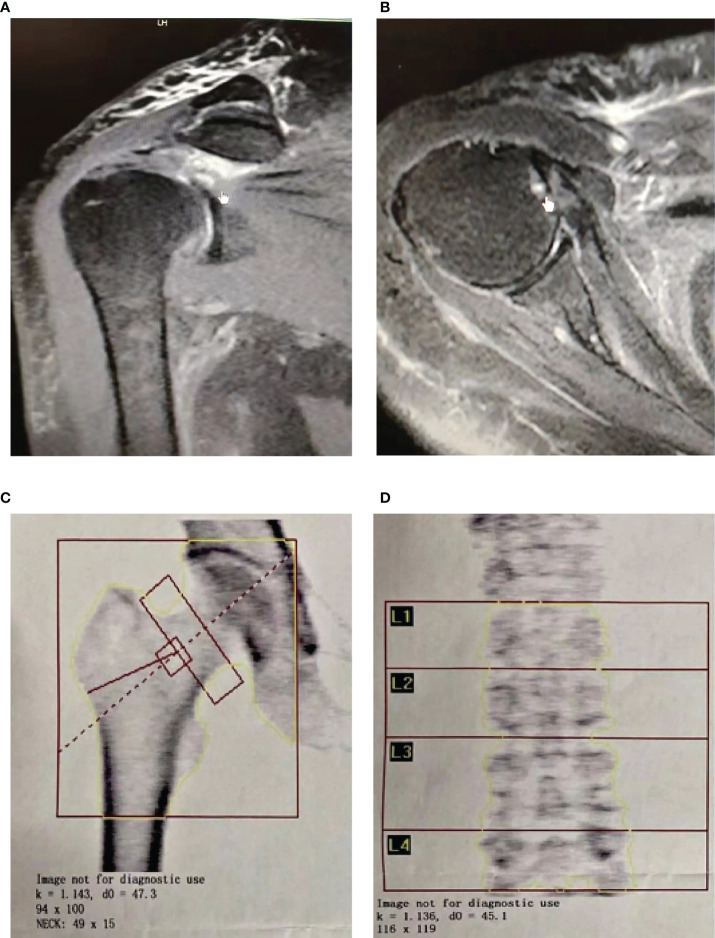
Rotator cuff tear with osteoporosis patients were defined by MRI **(A, B)** and DXA **(C, D)**.

In **Table 1** and **Figure 1**, a total of 104 cases were included in our study. Among them, there were 72 cases of RCTs-OP patients, 32 cases of RCTs patients, 14 diabetic patients in the selected population, 38 patients with hypertension, 4 cases of cerebral infarction patients, and 38 cases of other heart diseases. There were no statistically significant differences in patient basic information between the RCTs-OP and RCTs groups. All data on patients with training and validation cohorts include demographic, and the disease situation is indicated in **Supplement Tables 1**–**3**.

**Table 1:** Demographic and clinical characteristic.

Characteristic	RCTs (N=32)	RCTs-OP group (N=72)	All group (N=104)	P value
Female,n(%)	18 (33.3)	36 (66.70)	54 (51.92)	0.56
Age(y)	54.36 ± 11.86	54.88 ± 12.25	54.52 ± 11.92	0.48
BMI(cm2/kg)	24.38 ± 3.06	24.23 ± 3.53	24.33 ± 3.19	0.83
Weight(kg)	67.23 ± 10.14	66.59 ± 10.21	67.03 ± 10.11	0.76
Height(cm)	165.94 ± 6.75	165.81 ± 7.49	165.90 ± 6.95	0.92
Operative time(min)	45.25 ± 2.65	41.68 ± 5.69	43.61 ± 4.61	0.25
Right side,n(%)	14 (25.9%)	40 (74.1)	54 (51.92)	0.26
Diabete,n(%)	2 (6.3)	12 (16.7)	14 (13.5)	0.15
Hypertension,n(%)				
0	18 (56.25)	48 (66.67)	66 (64.71)	0.39
1	10 (31.25)	18 (25.00)	28 (27.45)	0.33
2	4 (12.5)	4 (5.56)	8 (7.69)	0.44
3	0(0)	2 (6.25)	2 (1.92)	0.12
other Heart disease,n(%)	14 (43.8)	24 (33.3)	38 (36.5)	0.31
Stoke,n(%)	2 (6.3)	2 (2.8	4 (3.8)	0.39

In **Table 2**, we also analyzed bone density results and results for bone metabolism markers. The results of bone density and T-values were first analyzed for the lumbar spine, right hip, and femoral neck. The results found significant differences between the two groups. At the same time, we also analyzed the results of bone metabolism markers and found that the levels of 25-VitD and OCN in the RCTs-OP group were significantly higher than those in the normal group.

**Table 2:** Bone metabolism index between two group.

Characteristic	RCTs (N=32)	RCTs-OP group (N=72)	All group	P value
Lumber_BMD (g/cm2)	1.02 ± 0.12	0.81 ± 0.10	0.95 ± 0.15	<0.001
Right_hip BMD (g/cm2)	0.96 ± 0.10	0.75 ± 0.07	0.89 ± 0.13	<0.001
Fummer BMD (g/cm2)	0.81 ± 0.11	0.65 ± 0.08	0.76 ± 0.13	<0.001
Lumber_T score	&-0.44 ± 1.22	&-2.12 ± 0.92	&-1.02 ± 1.42	<0.001
Right_hip_T score	&-0.32 ± 0.65	&-2.03 ± 0.68	&-0.64 ± 0.99	<0.001
Fummer_T score	&-0.69 ± 0.91	&-1.96 ± 0.54	&-1.08 ± 1.01	<0.001
Serum Ca (mmol/L)	2.41 ± 0.09	2.36 ± 0.09	2.37 ± 0.09	0.025
Serum P (mmol/L)	1.08 ± 0.17	1.08 ± 0.15	1.08 ± 0.15	0.821
25-VitD (nmol/L)	24.92 ± 10.21	22.20 ± 10.86	22.73 ± 10.64	0.015
ALP (U/L)	119.25 ± 24.28	118.70 ± 30.10	118.87 ± 28.32	0.928
Calcitonin (ng/ml)	8.45 ± 3.36	7.56 ± 4.13	7.83 ± 3.91	0.283
OCN (ug/L)	14.43 ± 5.95	13.25 ± 4.16	13.61 ± 4.78	0.026
CTX (ng/ml)	0.39 ± 0.22	0.33 ± 0.14	0.35 ± 0.166	0.907
PTH (g/ml)	26.54 ± 11.39	28.31 ± 10.03	27.76 ± 10.45	0.028

In **Table 3**, we analyzed serum amino acid levels, ornithine and diaminocaproic acid levels increased significantly in the RCTs-OP group, but γ_aminobutyric_acid showed a significant decrease. In **Tables 4**, **5** we analyzed the levels of vitamins in the serum and found that the levels of vitamin B12, vitamin D3, and vitamin E decreased significantly in the RCTs-OP group. However, vitamin C levels have increased significantly in the RCTs-OP group than Sham group.

**Table 3:** Serum amino acid between two group.

Characteristic	RCTs (N=32)	RCTs-OP group (N=72)	All group	P value
Glycine (umol/L)	212.91 ± 49.05	219.19 ± 43.77	214.85 ± 47.37	0.535
Serine (umol/L)	125.58 ± 26.98	121.97 ± 28.24	124.45 ± 27.30	0.538
Asparagine (umol/L)	55.92 ± 10.72	59.20 ± 13.51	56.93 ± 11.68	0.187
Arginine (umol/L)	47.72 ± 28.13	50.04 ± 33.34	48.44 ± 29.68	0.716
Alanine (umol/L)	537.95 ± 190.49	485.56 ± 168.52	521.83 ± 184.80	0.183
Citrulline (umol/L)	34.77 ± 9.95	34.38 ± 10.00	34.65 ± 9.92	0.853
Aminobutyric_acid (umol/L)	21.03 ± 5.13	21.31 ± 4.52	21.12 ± 4.93	0.797
Proline (umol/L)	221.86 ± 77.65	219.63 ± 93.18	221.17 ± 82.29	0.899
Valine (umol/L)	260.50 ± 61.58	244.62 ± 50.49	255.61 ± 58.62	0.204
Methionine (umol/L)	26.07 ± 5.99	26.16 ± 8.52	26.09 ± 6.83	0.947
Tyrosine (umol/L)	58.98 ± 13.52	57.64 ± 12.74	85.57 ± 13.24	0.635
Ornithine (umol/L)	136.75 ± 30.24	120.89 ± 22.89	131.87 ± 29.02	0.009
Phenylalanine (umol/L)	65.67 ± 12.59	68.34 ± 12.58	66.49 ± 12.59	0.322
Isoleucine (umol/L)	73.80 ± 34.80	79.05 ± 31.48	75.42 ± 33.75	0.467
Leucine (umol/L)	139.56 ± 43.17	142.30 ± 40.00	140.40 ± 42.05	0.76
Hydroxypuranine (umol/L)	13.34 ± 5.68	14.75 ± 5.89	13.77 ± 5.76	0.251
Threonine (umol/L)	123.33 ± 34.27	132.50 ± 48.48	126.15 ± 39.18	0.272
Tryptophan (umol/L)	83.81 ± 27.34	83.00 ± 25.94	83.56 ± 26.80	0.888
Glutamic_acid (umol/L)	153.61 ± 29.88	155.78 ± 40.02	154.27 ± 33.15	0.759
Aspartic_acid (umol/L)	9.60 ± 2.92	9.86 ± 1.55	9.68 ± 2.58	0.636
Sarcosine (umol/L)	1.36 ± 0.69	1.19 ± .48	1.34 ± 0.64	0.211
Histidine (umol/L)	94.05 ± 12.73	93.41 ± 11.76	93.85 ± 12.39	0.609
Methylhistine (umol/L)	3.88 ± 1.41	3.59 ± 1.62	3.80 ± 1.48	0.347
Diaminocaproic_acid (umol/L)	184.17 ± 39.31	163.68 ± 39.92	177.87 ± 40.43	0.016
3_Methylhistamine (umol/L)	2.63 ± 2.70	1.73 ± 1.33	2.35 ± 2.39	0.076
Aminoglycolic_acid (umol/L)	1.11 ± 0.52	1.01 ± 0.24	1.08 ± 0.46	0.328
3_Aminoisobutyric_acid (umol/L)	2.02 ± 1.14	2.26 ± 1.12	2.09 ± 1.13	0.321
Homogeneous_proline (umol/L)	1.02 ± 0.47	0.88 ± 0.45	0.98 ± 0.46	0.176
γ_aminobutyric_acid (umol/L)	0.05 ± 0.01	0.09 ± 0.20	0.06 ± 0.12	0.041
β_Alanine (umol/L)	3.14 ± 1.23	3.38 ± 1.81	3.22 ± 1.43	0.432

**Table 4:** Serum vitamin between two group.

Characteristic	RCTs (N=32)	RCTs-OP group (N=72)	All group	P value
Vitamin_B1 (ng/ml)	3.41 ± 1.79	3.17 ± 1.30	3.34 ± 1.65	0.486
Vitamin_B2 (ng/ml)	8.63 ± 5.05	7.87 ± 2.92	8.39 ± 4.50	0.423
Niacin_B3 (ng/ml)	28.38 ± 16.90	25.90 ± 16.85	27.62 ± 16.84	0.409
Pantothenic_acid (ng/ml)	52.58 ± 20.00	51.70 ± 19.65	52.31 ± 19.80	0.837
Vitamin_B6 (ng/ml)	6.95 ± 5.63	8.36 ± 7.95	7.39 ± 6.43	0.305
Biotin_B7 (ng/ml)	0.51 ± 0.17	0.46 ± 0.12	0.49 ± 0.15	0.145
folacin (ng/ml)	15.19 ± 14.64	17.65 ± 17.47	15.95 ± 15.52	0.458
Vitamin_B12 (ng/ml)	0.79 ± 0.65	0.59 ± 0.22	0.73 ± 0.56	0.001
Vitamin_C (ug/ml)	9.78 ± 2.99	10.03 ± 2.50	9.86 ± 2.83	0.034
Vitamin_A (ng/ml)	498.49 ± 130.34	505.54 ± 136.90	500.70 ± 131.79	0.803
Vitamin_d2 (ng/ml)	1.68 ± 3.78	0.75 ± 0.42	1.39 ± 3.17	0.172
Vitamin_D3 (ng/ml)	19.60 ± 9.19	16.71 ± 4.91	18.71 ± 8.21	0.027
Vitamin_E (ug/ml)	11.79 ± 3.54	10.08 ± 3.78	11.26 ± 3.69	0.028
Vitamin_K1 (ng/ml)	1.88 ± 2.62	1.19 ± 0.82	1.67 ± 2.24	0.144
Vitamin_D (ng/ml)	22.11 ± 10.03	19.33 ± 7.97	21.25 ± 9.49	0.107
Proalbumin (ng/ml)	292.89 ± 44.68	290.75 ± 45.63	292.23 ± 44.76	0.823
iron (ng/ml)	22.10 ± 7.16	21.48 ± 8.30	21.91 ± 7.49	0.697
Total_iron_binding_force (ng/ml)	61.71 ± 5.67	61.10 ± 5.57	61.52 ± 5.62	0.611

**Table 5:** Result multivariate Logistic regression analysis of clinical factors.

Variable	Exp (B)	t	P value
L_BMD	-3.283	-12.562	0
PTH	-0.009	-4.554	0
Ca	1.942	8.297	0
Asparagine	0.023	9.406	0
serine	-0.008	-8.176	0
Aminoglycolic_acid	-0.36	-5.829	0
leucine	0.008	7.094	0
Vitamin_B6	0.028	7.182	0
valine	-0.004	-4.763	0
F_T	0.076	1.924	0.058
site	0.231	5.431	0
Vitamin D	0.26	4.577	0
Calcitonin	-0.025	-3.886	0
Vitamin_B12	-0.054	-3.555	0.001
Pantothenic_acid	0.003	2.45	0.016

### Feature selection

**Figure 2:**
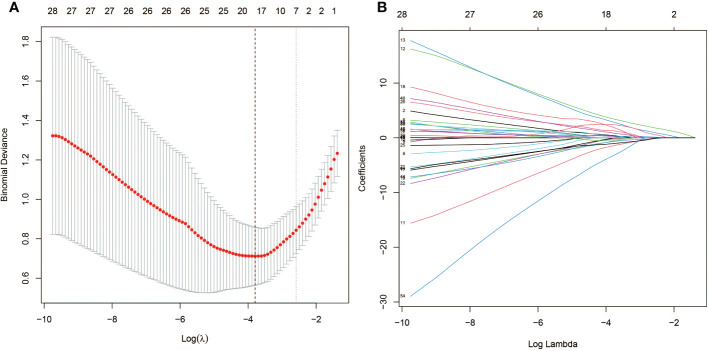
Demographic and clinical feature selection using the LASSO binary logistic regression model. **(A)** Optimal parameter (lambda) selection in the LASSO model used fivefold cross-validation *via* minimum criteria. **(B)** LASSO coefficient profiles of the 72 features. A coefficient profile plot was produced against the log(lambda) sequence. Vertical line was drawn at the value selected using fivefold cross-validation, where optimal lambda resulted in five features with nonzero coefficients.

Only four potential predictors were reduced from 72 features based on 76 patients in the training cohort and nonzero coefficients in the LASSO regression model were also built-in **Figure 2**. This feature included lumber T score, femur T score, Niacin B3, and Vitamin D (**Table 6**).

**Table 6:** RCT and OP risk prediction model.

Inrercept and variable	Prediction model		
	β	HR(95%CI)	P value
intercept	-2.53	0.079 (0.000426,6.99)	0.36
L_T	5.724225	30.61958 (1.204531,185.2538)	<0.001
F_T	4.696673	10.95820 (5.505512,118.0977)	<0.001
Niacin_B3	-5.43004	0.004382911 (0.0002049760,0.4503745)	0.03
Vitamin_D	-3.44031	0.03205484 (0.0008665031,0.3632817)	0.02

### Nomogram development and internal validation

**Figure 3:**
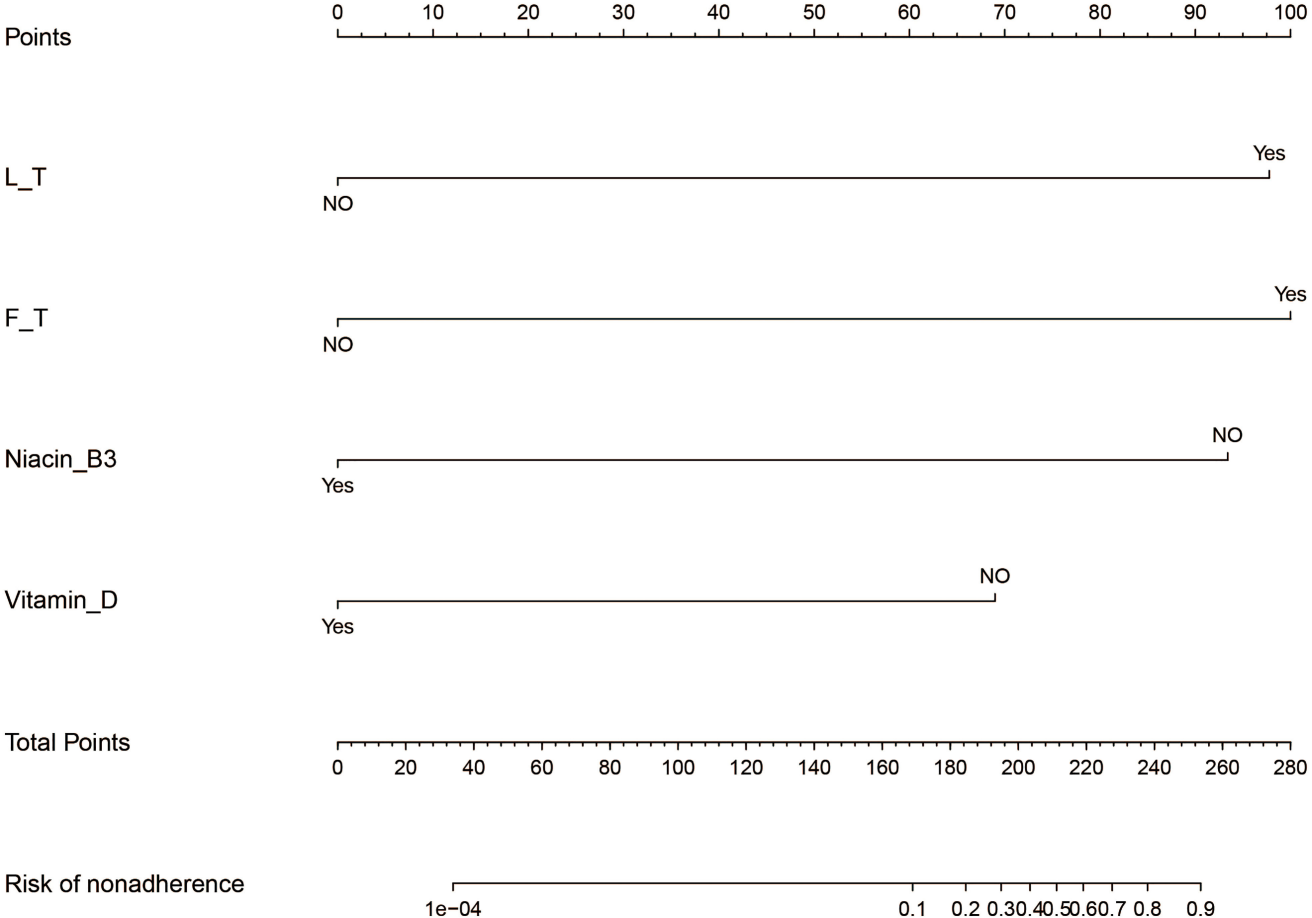
Developed prediction nomogram.

**Figure 4:**
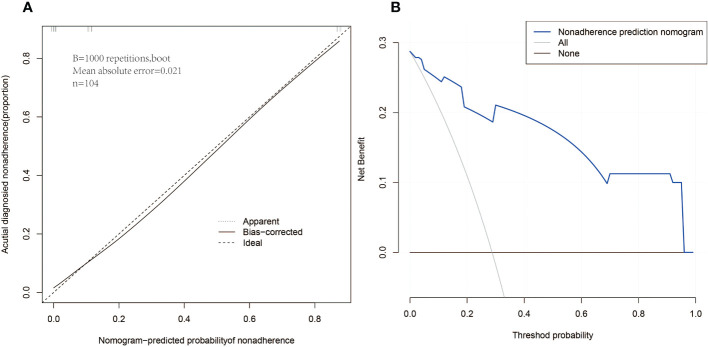
Calibration curves **(A)** and decision curve analysis **(B)** for nomogram in cohort.

Logistic regression analysis using the above four factors such as lumber T score, femur T score, Niacin B3, and Vitamin D have been indicated in **Table 6**. Models with the above independent predictors have been developed and presented in the form of nomograms (**Figure 3**). The risk calibration curve for the non-concordant isotype map (**Figure 4**) showed good agreement in this group of patients (**Figure 4**). The nomogram C-index predicted by the training group was 0.938 (95% CI: -1.83 to 1.39), and the initiation control C-index was 0.95, indicating good discrimination. The breach risk graph shows good predictability.

### Clinical application

**Figure 5:**
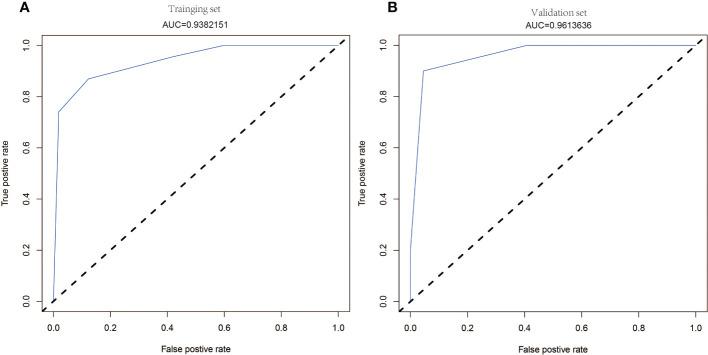
The ROC curves of the nomogram in traning and validation sets, respectively. The ROC curves of the nomogram are outperformed than the clinlical signature in both the training **(A)** and validation **(B)** sets.

The decision curve analysis for osteoporosis and rotator cuff injury is shown in **Figure 4**. Decision curves suggest that the use of this prediction nomogram to predict the risk of osteoporosis-related rotator cuff injury is more favorable than this protocol. When the threshold patient and physician probabilities were more than equal to 14 and less than 88%, respectively. Within this range, the net benefit was comparable with several overlaps, based on the pediction nomogram. ROC curves of the RCTs and RCTs-OP signature is shown in **Figure 5**. The AUC of the osteoporosis associated with rotator cuff injury nomogram was higher than that of the clinical factor model (p = 0.035, AUC=0.938) in the training set and there was a significant difference in the AUC between the osteoporosis associated with rotator cuff injury nomogram and clinical factors model in the validation set (p = 0.012, AUC=0.961).

### Correlation between serum vitamin D level with baseline characteristic of RCTs-OP patients

The results of the clinical predictive model found that vitamin D is an independent predictive shadow that affects rotator cuff injury with osteoarthritis, so we further use vitamin D as a basis for further grouping. Patients with RCTs-OP were subdivided into high- vitamin D and low vitamin D group (**Table 7**). As shown in **Table 7**. The patients with high vitamin D group have low risk in stoke than patients with low vitamin D level. In addition, the OCN, CTX, alanine, Glutamic_acid, 3_Aminoisobutyric_acid, Vitamin_B1, Vitamin_B6, Vitamin_A in high vitamin D group than low vitamin D group but phenylalanine, Sarcosine, Biotin_B7, Vitamin_E and Vitamin_K1 were low in high vitamin D group than low vitamin D group.

**Table 7:** Comparison of serum VitD levels with baseline characteristics of RCTs-OP patients.

Characteristics	Low Vit D group (54)	High Vit D group (18)	P value
Female,n (%)	30 (55.56)	11 (61.11)	0.105
Age (y)	54.44 ± 13.23	54.11 ± 6.41	0.919
BMI (cm2/kg)	24.06 ± 2.88	25.33 ± 3.48	0.132
Weight (kg)	66.36 ± 10.42	69.86 ± 8.98	0.207
Height (cm)	165.81 ± 6.81	166.33 ± 6.74	0.78
Diabete,n (%)	8 (14.81)	2 (11.11)	0.472
Hypertension,n (%)	9 (16.67)	2 (11.11)	0.459
Stoke,n (%)	8 (14.81)	3 (16.67)	0.013
Lumber_BMD (g/cm2)	1.00 ± 0.13	1.06 ± 0.13	0.093
Right_hip BMD (g/cm2)	0.94 ± 0.11	0.99 ± 0.09	0.094
Fummer BMD (g/cm2)	0.87 ± 0.11	0.82 ± 0.09	0.781
Lumber_T score	&-0.58 ± 1.17	&-0.11 ± 1.27	0.086
Right_hip_T score	&-0.28 ± 0.76	&0.14 ± 0.91	0.052
Fummer_T score	&-0.76 ± 0.89	&-0.46 ± 0.96	0.242
Serum Ca (mmol/L)	2.36 ± 0.08	2.33 ± 0.13	0.126
Serum P (mmol/L)	1.07 ± 0.15	1.11 ± 0.15	0.365
ALP (U/L)	115.67 ± 31.09	127.78 ± 25.56	0.141
Calcitonin (ng/ml)	7.58 ± 4.43	7.47 ± 3.13	0.916
OCN (ug/L)	12.70 ± 4.40	14.90 ± 2.81	0.018
CTX (ng/ml)	0.30 ± 0.12	0.42 ± 0.15	0.001
PTH (g/ml)	29.31 ± 10.61	25.28 ± 7.51	0.142
Glycine (umol/L)	206.39 ± 46.86	232.46 ± 51.59	0.05
Serine (umol/L)	129.08 ± 27.99	115.48 ± 21.44	0.065
Asparagine (umol/L)	55.83 ± 11.72	56.18 ± 7.19	0.906
Arginine (umol/L)	46.44 ± 29.16	51.59 ± 25.14	0.504
Alanine (umol/L)	491.55 ± 175.09	677.15 ± 169.21	0.001
Citrulline (umol/L)	34.31 ± 10.86	36.16 ± 6.55	0.498
Aminobutyric_acid (umol/L)	21.041 ± 5.02	21.01 ± 5.60	0.984
Proline (umol/L)	223.49 ± 84.07	216.95 ± 55.80	0.759
Valine (umol/L)	259.15 ± 61.76	264.53 ± 62.64	0.751
Methionine (umol/L)	26.52 ± 6.12	24.67 ± 5.53	0.259
Tyrosine (umol/L)	60.59 ± 13.72	54.15 ± 11.97	0.08
Ornithine (umol/L)	138.41 ± 32.98	131.75 ± 19.82	0.422
Phenylalanine (umol/L)	67.15 ± 13.25	61.23 ± 9.33	0.044
Isoleucine (umol/L)	76.77 ± 36.23	64.88 ± 29.21	0.212
Leucine (umol/L)	138.73 ± 42.78	142.02 ± 45.49	0.781
Hydroxypuranine (umol/L)	12.63 ± 5.94	15.46 ± 4.28	0.034
Threonine (umol/L)	125.03 ± 37.95	118.22 ± 19.44	0.469
Tryptophan (umol/L)	81.13 ± 28.47	91.83 ± 22.45	0.152
Glutamic_acid (umol/L)	148.85 ± 31.05	167.87 ± 20.90	0.018
Aspartic_acid (umol/L)	9.63 ± 3.30	9.51 ± 1.23	0.879
Sarcosine (umol/L)	1.49 ± .70	.96 ± .52	0.004
Histidine (umol/L)	93.71 ± 12.89	95.07 ± 12.50	0.695
Methylhistine (umol/L)	3.95 ± 1.58	3.68 ± .64	0.491
Diaminocaproic_acid (umol/L)	182.51 ± 40.64	189.15 ± 35.60	0.538
3_Methylhistamine (umol/L)	2.82 ± 2.96	2.08 ± 1.67	0.324
Aminoglycolic_acid (umol/L)	1.14 ± .58	.99 ± .25	0.302
3_Aminoisobutyric_acid (umol/L)	1.80 ± .87	2.65 ± 1.54	0.005
Homogeneous_proline (umol/L)	1.01 ± .52	1.04 ± 0.27	0.822
γ_aminobutyric_acid (umol/L)	.05 ± .01	.04 ± .01	0.037
β_Alanine (umol/L)	3.25 ± 1.21	2.84 ± 1.29	0.226
Vitamin_B1 (ng/ml)	3.03 ± 1.35	4.55 ± 2.40	0.001
Vitamin_B2 (ng/ml)	7.96 ± 4.08	10.61 ± 7.00	0.053
Niacin_B3 (ng/ml)	28.56 ± 19.13	27.83 ± 7.13	0.875
Pantothenic_acid (ng/ml)	54.05 ± 19.82	48.14 ± 20.41	0.281
Vitamin_B6 (ng/ml)	5.99 ± 4.89	9.81 ± 6.77	0.012
Biotin_B7 (ng/ml)	.53 ± .15	.41 ± .15	0.007
folacin (ng/ml)	15.04 ± 15.76	15.64 ± 10.97	0.881
Vitamin_B12 (ng/ml)	.85 ± .73	.60 ± .21	0.859
Vitamin_C (ug/ml)	9.32 ± 2.87	11.12 ± 2.96	0.16
Vitamin_A (ng/ml)	472.58 ± 123.02	585.92 ± 118.74	0.026
Vitamin_d2 (ng/ml)	1.89 ± 4.34	1.02 ± .42	0.122
Vitamin_D3 (ng/ml)	17.30 ± 8.81	26.49 ± 6.63	0.402
Vitamin_E (ug/ml)	12.35 ± 3.74	10.11 ± 2.20	0.001
Vitamin_K1 (ng/ml)	2.11 ± 2.96	1.21 ± .77	0.003
Proalbumin (ng/ml)	291.52 ± 47.952	297.00 ± 33.82	0.655
iron (ng/ml)	21.54 ± 7.82	23.75 ± 4.41	0.26
Total_iron_binding_force (ng/ml)	61.32 ± 6.13	62.86 ± 3.85	0.07

Furthermore, while a correlation was established between vitamin D with baseline characteristic of RCTs-OP patients. It is found that the serum vitamin D were negative correlated with Hyertension, diabete, serine, proline, isoleucine, threonine and biotin B7 but positive correlated with CTX, 3 Aminoisobutyric acid, Vitamin B1, Vitamin B2, Vitamin_B6, Vitamin C and Vitamin A (**Supplement Table 4**). This further suggests that vitamin D level was an independent factor in those with RCTs-OP patients.

## Discussion

Rotator cuff repair surgery in patients with osteoporosis is now receiving increasing attention, and anti-osteoporosis treatment has shown significant results in improving rotator cuff repair surgery (38). There is a clear association between osteoporosis and rotator cuff injury in clinical studies. In previous studies, rotator cuff injuries patients with osteoporosis have a 1.79 times higher risk than those patient without osteoporosis (39). Aiming to establish a prognostic system independent of clinical characteristics, bone metabolism marker, amino acid, and serum vitamin. We assessed multiple clinical characteristics, bone metabolism marker, amino acid, and serum vitamin closely related to host RCTs related OP status. This findings of this study demonstrate that OP with RCTs has a lower level of in 25-vitD, OCN, serum Ca^2+^,ornithine, diaminocaproic_acid but the high level of γ_aminobutyric_acid, Vitamin_C, Vitamin_D3 and Vitamin_E, Vitamin_B12, than RCTs patients without OP. In addition. the prediction model for risk among RCTs with OP merely used four easily available variables and appeared more effective clinical response index. At the same time, the use of training cohorts and validation cohorts further improved the accuracy of prognostic indicators. Finally, Multivariable logistic regression revealed that low serum VitD level was an independent risk factor for RCTs-OP patients. Thus, vitamin D level may be valuable prognostic biomarkers that reflect the combined effect of vitamins on the progression of RCT-related OP.

Nomograms are widely used as diagnostic methods in oncology and medicine which can manifest as very friendly interfaces, accuracy, and ease of understanding to help make clinical diagnoses (40, 41). Our study is the first to study the relationship between rotator cuff injury and osteoporosis using the nomograms method. Addition of risk factors for demographic, pathological, and treatment characteristics into an easy-to-use nomogram facilitates individual prediction of surgical risk using RCTs. Finally, the internal cohort validation showed good discriminative strength and calibration efficiency; in particular, the c-index was high for range validation, indicating that the nomogram has a large sample size and can be used widely and accurately (40, 42). The multivariate Logistic regression analysis has been carried out with clinical factors and it also found that femur T score, Vitamin D and vitamin B12 have significantly different between RCTs with OP or without OP. Data on prevalence and identification of underlying risk factors facilitate early diagnosis of randomized controlled trials and improve treatment outcomes (43).

During the healing process of tendon bone, obvious bone mass loss was found in the area, including the proximal tibia of the anterior cruciate ligament and the calcaneal bone after Achilles tendon injury (44, 45). Osteoporosis increases the revision rate of rotator cuff repair due to lower bone mass at the insertion of the humeral cephalic tendon (46). Consistent with the literature, this research found that participants who reported rotator cuff injuries have consistent changes in BMD and T-values of the lumbar spine, femoral head, and humerus. This means that proximal humerus BMD is highly reliable and can be accurately assessed preoperatively in patients requiring suture anchors for rotator cuff repair and in patients with proximal humerus fractures (47). Besides, Osteoporosis affects the healing of the rotator cuff, and the possible cause is related to the fact that high osteoclast activity can lead to poor healing of the injury interface in the early stages (39, 48). However, in our study, Indicators of osteoclast activity in serology did not show significant changes in patients with rotator cuff injury with osteoporosis which may be associated with increased local osteoclast activity but insignificant serologic osteoclast activity changes.

PTH and OCN are also important diagnostic markers of osteoporosis. In our study, it was also found that patients with rotator cuff injuries with osteoporosis experience changes in OCN and PTH levels. A close association between PTH and OCN and rotator cuff injury was also found in multiple regression models. Yoon et al.found that Local administration of PTH *via* an absorbable scaffold improves biomechanical and histological treatment of rotator cuff in rat tendons (49). Lower levels of osteocalcin have also been found in tenocyte-like cells from the human rotator cuff which are in line with those of our studies (50).

Vitamins D are essential constituents of our diet that have long been known to influence human health (51). Preliminary data suggest that vitamins D can promote tendon growth and healing and improve bone mass. Serum vitamin D levels were significantly negatively correlated with cuff steatosis and significantly positively correlated with isokinetic muscle circulation (52). Vitamin D insufficiency was associated with rotator cuff muscle strength in professional volleyball athletes (11). Jourdan et al. investigated Association between serum 25-hydroxyvitamin D levels and failure of arthroscopic rotator cuff repair and statistically significant association between serum 25-hydroxyvitamin D deficiency and rotator cuff deficiency and frequency of controls (53). In addition, Vitamin B12 is also closely associated with rotator cuff injuries. Vitamin B12 deficiency can lead to high levels of homocysteine, which interferes with collagen cross-linking, thereby compromising tendon integrity (13). Chi et al. found that Vitamin B12, vitamin D and angiopoietin are significantly reduced in tendon disorders, which may contribute to a better understanding of the complex pathology of tendon disorders (54). High vitamin D level also promote bone health (55). Garicia et al.also found that associations between 25(OH)D concentrations during fetal life with BMC and bone area in childhood, but these associations were no longer significant after adjustment for childhood 25(OH)D status (56). What’s more, poor vitamin D status is common in patients with impaired renal function and represents one main component of the complex scenario of chronic kidney disease-mineral and bone disorder (57). Vitamin D status also Established risk factors for osteoporosis and associated fractures (58). Cochrane review investigated that Vitamin D and related compounds have been used to prevent osteoporotic fractures in older people (59). consensus statement from the European Society for Clinical and Economic Aspects of Osteoporosis and Osteoarthritis (ESCEO) found that vitamin D play important role in maintaining musculoskeletal health in postmenopausal women (60). Whether vitamin D levels also have a clear effect on rotator cuff injury and osteoporosis is an important issue in our study. Therefore, more research on tendon health and nutrition is needed, possibly because tendon health requires more nutrients and a multi-nutrient intervention may be more effective than a single nutritional strategy.

Amino acid levels play an important role in tendon-bone healing which act as another important nutrient. The addition of amino acids to glutamine, arginine, and branched-chain amino acids (leucine, isoleucine, and valine) significantly increased skin collagen synthesis in UV-irradiated mice (61). Several studies have shown that dietary intake of leucine stimulates protein synthesis in mammals through the rapamycin signaling pathway (62). Other studies have shown that oral addition of a mixture of methyl beta-hydroxy-beta-butyrate, glutamine, and arginine can significantly increase muscle mass and muscle mass, or both, even in older adults. in this way (63). These results support our study that leucine or other nutrients can increase tenascin synthesis and function. Therefore, the lack of understanding of the relationship between diet and tenascin requires extensive research in this field (64, 65).

Our study developed a novel nomogram with relatively good accuracy to help physicians understand osteoporosis risk in RCT patients, including clinical features, markers of bone metabolism, amino acids, and serum vitamins. By assessing individual risk, surgeons and patients can conduct BMD monitoring and medical interventions where they are most needed. This nomogram requires external validation, and more research is needed to determine whether individualized interventions based on this nomogram can improve treatment outcomes.

## Limitations

There are some limitations in interpreting our results. First, the retrospective nature of the study design was limited by the accuracy of the data stored in the graphs. At the time of data collection, the Department of Sports Medicine had no formal osteoporosis screening program; therefore, these numbers may not reflect other institutions that use screening programs. Second, the study did not examine clinical outcomes in healthy people or the effect of bone health on outcomes. Finally, these figures reflect the number of patients in the geographic region of China. It is unclear whether these data reflect patient populations in other regions.

## Conclusion

The main goal of the current study was to determine the relationship between RCTs and OP and found that vitamin D are valuable prognostic biomarkers, reflecting the combined effect of vitamins on RCTs related OP progression. These findings also highlight that nutrients condition such as vitamins and amino acids of patients provide a new understanding of the development of RCTs.

## Data availability statement

The original contributions presented in the study are included in the article/**Supplementary Material**. Further inquiries can be directed to the corresponding authors.

## Ethics statement

This study was reviewed and approved by the ethics committee and the institutional review board (IRB) of The Shanghai Tenth People’s Hospital of Tongji University (SHSY-IEC-KY-4.0/19-05/01). Written informed consent was obtained from the owners for the participation of their animals in this study.

## Author contributions

Conception and design: GJL, WJL. Acquisition, analysis, and interpretation of the data: GJL, WJL, LYZ, CZ. Drafting and writing: CZ, RJC. Final approval of the article: GJL, WJL, LYZ, CZ, RJC. All authors contributed to the article and approved the submitted version.

## Funding

This research was funded by Clinical research project of Shanghai Tenth People’s Hospital (Grants no. YSDDSD11W).

## Acknowledgments

We would like to thank all participants and our hospital.

## Conflict of interest

The authors declare that the research was conducted in the absence of any commercial or financial relationships that could be construed as a potential conflict of interest.

## Publisher’s note

All claims expressed in this article are solely those of the authors and do not necessarily represent those of their affiliated organizations, or those of the publisher, the editors and the reviewers. Any product that may be evaluated in this article, or claim that may be made by its manufacturer, is not guaranteed or endorsed by the publisher.
