# Supplementary material for: The role of vitamin D on rotator cuff tear with osteoporosis
Source: Front Endocrinol (Lausanne). 2022 Nov 18;13:1017835. doi: 10.3389/fendo.2022.1017835 (PMC9716320; doi:10.3389/fendo.2022.1017835)
Supplement: Supplementary file 1 [file DataSheet_1.docx]

| Supplement Table1. clincial characteristic between training and validation sets | | | | | | |
| --- | --- | --- | --- | --- | --- | --- |
|  | Trainging set(76) | | | Validation set(n=28) | | |
| Characteristic | RCTs(N=24) | RCTs-OP (N=52) | P value | RCTs(N=8) | RCTs-OP(N=20) | P value |
| Female,n(%) | 11(45.8) | 26(50.0) | 0.736 | 3(37.5) | 10(50) | 0.549 |
| Age(y) | 54.50±12.68 | 55.37±10.77 | 0.759 | 56.00±11.58 | 51.75±14.29 | 0.465 |
| BMI(cm2/kg) | 24.46±3.14 | 24.57±3.18 | 0.895 | 23.3±4.71 | 23.89±2.75 | 0.801 |
| Weight(kg) | 68.19±9.39 | 67.89±10.55 | 0.908 | 61.81±11.71 | 65.51±9.00 | 0.375 |
| Height(cm) | 167.00±7.64 | 166.10±6.75 | 0.604 | 162.25±2.07 | 165.55±6.89 | 0.248 |
| Right side,n(%) | 11(59.6) | 31(45.8) | 0.261 | 3(37.5) | 9(45.0) | 0.717 |
| Diabete,n(%) | 1(4.2) | 9(17.3) | 0.115 | 1(12.5) | 3(15) | 0.864 |
| Hypertension,n(%) |  |  |  |  |  |  |
| 0 | 13(54.2) | 33(63.5) | 0.719 | 5(62.5) | 15(75.0) | 0.378 |
| 1 | 8(33.3) | 14(26.9) |  | 2(25.0) | 4(20) |  |
| 2 | 3(12.5) | 4(7.7) |  | 1(12.5) | 0(0) |  |
| 3 | 0(0) | 1(1.9) |  | 0(0) | 1(5) |  |
| other Heart disease,n(%) | 14(58.3) | 10(41.7) | 0.554 | 4(50) | 6(30) | 0.318 |
| Stoke,n(%) | 1(4.2) | 1(1.9) | 0.57 | 1(12.5) | 1(5) | 0.486 |
| Lumber_BMD(g/cm2) | 1.03±0.13 | 0.82±0.10 | **<0.001** | 0.99±0.10 | 0.76±0.10 | **<0.001** |
| Right_hip BMD(g/cm2) | 0.97±0.11 | 0.76±0.07 | **<0.001** | 0.92±0.08 | 0.75±0.07 | **<0.001** |
| Fummer BMD(g/cm2) | 0.82±0.11 | 0.65±0.08 | **<0.001** | 0.77±0.09 | 0.64±0.08 | **<0.001** |
| Lumber_T score | &-0.35±1.26 | &-2.19±0.90 | **<0.001** | &-0.66±1.11 | &-2.71±0.93 | **<0.001** |
| Right_hip_T score | &-0.04±0.88 | &-1.65±0.36 | **<0.001** | &-0.54±0.48 | &-1.74±0.63 | **<0.001** |
| Fummer_T score | &-1.57±0.98 | &-1.94±.55 | **<0.001** | &-0.99±0.63 | &-2.00±0.57 | **0.001** |
| Serum Ca(mmol/L) | 2.42±0.09 | 2.35±0.10 | 0.109 | 2.38±0.11 | 2.37±0.10 | 0.982 |
| Serum P(mmol/L) | 1.08±0.15 | 1.07±0.15 | 0.917 | 1.12±0.22 | 1.10±0.16 | 0.778 |
| 25-VitD(nmol/L) | 24.23±10.61 | 22.32±10.08 | 0.421 | 22.98±9.50 | 21.90±12.94 | 0.832 |
| ALP(U/L) | 119.42±24.30 | 117.09±31.86 | 0.751 | 118.75±25.88 | 122.90±25.21 | 0.699 |
| Calcitonin(ng/ml) | 7.95±3.29 | 7.71±4.01 | 0.796 | 9.92±3.36 | 7.17±4.50 | 0.131 |
| OCN(ug/L) | 13.69±5.58 | 13.43±4.50 | 0.831 | 16.68±6.85 | 12.80±3.16 | 0.047 |
| CTX(ng/ml) | 0.38±0.21 | 0.34±0.15 | 0.318 | 0.42±0.24 | 0.31±0.08 | 0.092 |
| PTH(g/ml) | 27.18±12.00 | 27.71±9.89 | 0.837 | 24.63±9.78 | 29.86±10.50 | 0.236 |

| supplement Table 4. The correlation of serum Vit D with other clinical indicators and osteoposis in RCTs patients | | |
| --- | --- | --- |
| Characteristics | Serum VitD | |
|  | r | P value |
| Hyertension | &-2.44 | **0.039** |
| Diabete | &-0.236 | **0.046** |
| CTX | 0.377 | **0.01** |
| Serine | &-0.286 | **0.017** |
| Proline | &-0.243 | **0.04** |
| Isoleucine | &-0.234 | **0.048** |
| Threonine | &-0.362 | **0.002** |
| 3_Aminoisobutyric_acid | 0.391 | **0.001** |
| Vitamin_B1 | 0.689 | **0.001** |
| Vitamin_B2 | 0.482 | **0.001** |
| Vitamin_B6 | 0.294 | **0.012** |
| Biotin_B7 | &-0.330 | **0.005** |
| Vitamin_C | 0.239 | **0.043** |
| Vitamin_A | 0.369 | **0.002** |
